# Form-Stable Phase-Change Materials Using Chemical Vapor Deposition-Derived Porous Supports: Carbon Nanotube/Diatomite Hybrid Powder and Carbon Nanotube Sponges

**DOI:** 10.3390/ma17235721

**Published:** 2024-11-22

**Authors:** Francesca Romana Lamastra, Mario Bragaglia, Lorenzo Paleari, Francesca Nanni, Francesco Fabborcino, Manuela Scarselli

**Affiliations:** 1 Dipartimento di Ingegneria dell’Impresa “Mario Lucertini”, Università degli Studi di Roma “Tor Vergata” and Consorzio INSTM Unità di Ricerca “Roma Tor Vergata”, Via del Politecnico, 00133 Roma, Italy; lamastra@scienze.uniroma2.it (F.R.L.); lorenzo.paleari@uniroma2.it (L.P.); fnanni@ing.uniroma2.it (F.N.); 2 Department of Engineering, Pegaso Telematic University, 80143 Naples, Italy; francesco.fabbrocino@unipegaso.it; 3 Dipartimento di Fisica, Università degli Studi di Roma “Tor Vergata”, Via della Ricerca Scientifica, 00133 Roma, Italy; manuela.scarselli@roma2.infn.it

**Keywords:** phase-change material, diatomite, carbon nanotubes

## Abstract

In this work, two types of chemical vapor deposition (CVD)-derived porous supporting materials consisting of CNTs–decorated diatomite (CNT/DE) and CNT sponges (CNS) were developed to prepare novel form-stable phase-change material (PCM) composites by impregnation, using polyethylene glycol (PEG) as the PCM. The CNT/DE support matrix showed highly entangled nanotubes (the weight ratio of CNTs to DE was 0.16) over and inside the porous structure of diatomite, giving the hybrid matrix an electrical response. The CNS that resulted was mainly composed of bent and interconnected CNTs forming a three-dimensional highly porous structure. XPS and FTIR results revealed that CNTs in both the supporting materials have a moderate amount of oxygen-containing functional groups. Both hosts allow for high PEG loading (about 75 wt%) without showing any PCM leakage during melting. Both form-stable PCM composites showed high thermal reliability upon a hundred melting–solidification DSC cycles (PEG/CNT/DE latent heat is 86 ± 4 J/g and PEG/CNS latent heat is 100 ± 2 J/g; melting temperature 34 °C). An analytical model was used to evaluate the passive cooling performance of the systems, simulating the thermal behaviour of a building wall containing the confined PCM in the hosts, resulting in a reduction in required cooling power of about 10%. The overall results suggest that the developed form-stable PCM composites could be considered promising additive materials for the production of building envelopes with thermal energy storage capability.

## 1. Introduction

The building and construction sector accounts for around one third of global energy use, contributing to 40% of global CO_2_ emissions [1]. Embodied energy (EE), i.e., total energy consumed to construct, maintain, and demolish a building, and operational energy (OE), i.e., energy consumed during the operation of a building, to maintain comfort and routine maintenance, including heating and cooling, space conditioning and ventilation, lighting, cooking, hot water provision and appliance use, are the two major components of the life cycle energy of buildings [2]. EE and OE roughly contribute to 10–20% and 80–90% of a building’s total life cycle energy, respectively [3]. Heating, ventilation and air conditioning systems represent about 38% of buildings’ OE and are responsible for about 12% of worldwide energy use [4]. Building envelope performance improvement, in terms of a smart management of the thermal energy flows from and to a building aimed at levelling out daily and seasonal temperature fluctuations, is critical to decrease heating and cooling demand [5,6]. Presently, the development of technologies to enhance energy efficiency in buildings, such as the incorporation of phase-change materials (PCMs) in building envelopes, has gained huge interest among researchers [5,7,8]. Based on their chemical composition, PCMs can be classified as organic, inorganic, and eutectic materials. Each category has a range of working temperatures and thermo-physical properties (i.e., phase-transition temperature, phase-transition enthalpy, and thermal conductivity) suitable for specific applications [9,10]. Organic PCMs are further classified as paraffin and non-paraffin: the latter include fatty acids, esters, alcohols and glycols [11,12,13]. PCMs with a melting/freezing temperature between 18 °C and 40 °C are particularly suitable for building applications [14]. Construction materials (i.e., concrete, mortars, cement) have an intrinsic sensible heat storage capacity between 0.75 and 1.00 J/g × K and low volumetric energy storage density [6,7]. The incorporation of PCMs in building envelopes, due to their high energy storage density in a narrow temperature interval (about 5–10 times higher compared to building elements, such as exteriors walls) results in achieving the overall regulation of indoor temperature regardless of external weather conditions and thus reducing the energy consumption of buildings [5,7,15]. PCMs are indeed capable of absorbing and releasing a large amount of thermal energy upon undergoing solid–liquid phase transitions, and when embedded in building components, they work as follows: (i) during the daytime, when heatwaves penetrate into the building envelopes, they reach melting point and absorb heat, resulting in delaying and/or lowering the peak of the heatwave within the building structure, and thus keeping the indoor temperature comfortable for most of the day; and (ii) at night, when the temperature falls below their phase transition temperature, the PCMs undergo solidification, releasing the stored heat to the interior and exterior of the building, and thus leading to a comfortable interior temperature [16]. Among the several techniques used to integrate PCMs into building components, micro/nano encapsulation and form stabilization are more effective in terms of heat transfer, preventing PCM leakage during melting, and corrosive resistance with respect to direct impregnation methods [7]. The form-stabilized PCM is a composite material, retaining the maximum amount of PCM and showing no leakage at melting temperatures [9]. The confinement of a PCM in a host (i.e., capsules, porous matrices, and layered materials) can be promoted by different interactions (i.e., capillary forces, covalent bonding, hydrogen bonding, and van der Waals forces), that can also act simultaneously and that depend on the PCM and host nature. The use of a form-stabilized PCM ensures reliability (i.e., PCM melting/solidification cycles can be repeated without degradation), which is a crucial feature for applications requiring long-term high performance, such as buildings [17]. Several form-stable PCM composites, consisting of PCMs incorporated into porous materials by means of immersion techniques, have been investigated in the literature [18,19,20,21,22]. Diatomite, also called diatomaceous earth (DE), is an amorphous silica-based mineral from the fossil residue of microscopic algae of the diatom family, composed mainly of diatom cell walls (frustules) exhibiting porous periodic structures ordered at the micro and nanoscale [23]. Diatomite, due to its unique properties such as high porosity (80–90%), thermostability, outstanding absorption capacity, chemical inertness, and relatively low cost [24,25], has been proved to be an ideal supporting material for PCMs [26,27,28,29,30]. Furthermore, since DE is widely used in building material mix design [31,32], it allows an easy integration of the incorporated PCM into such materials. The main drawback of both DE and PCMs for their application in thermal energy storage systems is their low thermal conductivity, resulting in slow heat transfer [33]. Carbon nanotubes (CNTs) have been successfully used to enhance the thermal conductivity of PCMs incorporated into diatomite [34]. As an example, Xu et al. [35] prepared a suspension of DE and CNTs in acetone, and after drying, the resulting mixture was impregnated with paraffin. Sari et al. [36] prepared DE/CNT pre-composites by (i) dispersing CNTs in acetone, (ii) adding DE and homogenizing the mixture by stirring and (iii) evaporating acetone in the pre-composites, and then (iv) the final composite was obtained incorporating the selected PCM (polyethylene glycol (PEG)) into the pre-composite using vacuum impregnation method. Also, surface modifications of diatomite based on the deposition of conductive nanoparticles (such as Cu and Ag nanoparticles) on its frustules have been proved to be effective strategies to improve the thermal conductivity of DE-based composite PCMs [37,38,39,40]. In the present paper, CNTs and CNSs were synthesized by a chemical vapor deposition (CVD) process. This technique offers some advantages in CNT and CNS synthesis compared to other preparation techniques, such as (i) scalability; (ii) tailored control of the carbon-based architecture; (iii) high purity; and (iv) cost effectiveness. It is the technique adopted at industrial level for the massive production of single- as well as multi-walled CNTs. It is essentially a two-step process consisting of a preliminary catalyst preparation followed by the actual synthesis of nanotubes. The catalyst is usually a transition metal, such as Ni, Fe, or Co, with high carbon solubility, that drives the CNT nucleation [41]. In this work, two different CVD-derived porous supports for the shape-stabilization of PCM were prepared. The first one was a porous hybrid matrix based on CNTs grown directly on DE, and the second one was a CNT sponge (CNS). PEG was employed as the PCM due to its relatively high latent heat storage capacity; suitable phase-change temperature range for application in building envelope, which can be tuned just by changing the molecular weight; isothermal phase transition behaviour; non-corrosiveness; non-toxicity; and chemical stability during melting/freezing processes [25,37,42]. PEG was confined in porous supports by impregnation, and the obtained form-stable composite PCMs were characterized from a chemical, thermal and morphological point of view. A numerical model was adopted to estimate the performances of an exterior building wall comprising a shape-stabilized PEG layer for passive cooling. To the best of the authors’ knowledge, a study focused on the development of form-stable PCMs using such supporting hosts has not yet been conducted.

## 2. Materials and Methods

### 2.1. Raw Materials

PEG (waxy solid, average molecular weight = 950–1050, melting point = 39 °C, Brookfield viscosity ≥ 20.0 cps) and DE (Celite^®^ S, powder composition: SiO_2_ = 90.2%, Al_2_O_3_ = 4.1%, CaO = 0.4%, Fe_2_O_3_ = 1.6%, MgO = 0.2%, Na_2_O + K_2_O = 1.4%, P_2_O_5_ = 0.3%, TiO_2_ = 0.2%, specific surface area 5–10 m^2^/g) were purchased from Sigma-Aldrich. Ferrocene (Fc, Sigma-Aldrich, St. Louis, MO, USA), 1,4-Dioxane (99% purity, Sigma-Aldrich, St. Louis, MO, USA), argon, hydrogen, and acetylene (standard purity) were used for the growth of CNTs on diatomite and preparation of CNS by CVD. 

### 2.2. Preparation of CNT/DE Host by CVD

CNTs were grown on diatomite following a CVD route. Ferrocene catalyst was added to DE by a wet method: (i) Fc (18.6 mg) was dissolved in 25 mL of ethanol into a flask kept in the dark, under mechanical stirring for 10 min at room temperature; (ii) DE (0.5 g), previously oven-dried at 80 °C for 24 h, was added to the fresh prepared solution of Fc and the obtained mixture was stirred for 30 min at 60 °C and then sonicated for 30 min in an ultrasound bath; (iii) the resulting powder (DE-Fc) was collected by vacuum filtration using a porcelain Buchner funnel with a filter paper placed on top, inserted in a vacuum flask connected to a vacuum pump. The collected DE-Fc powder was dried at 80 °C for 30 min for removing any ethanol traces and stored in an oven at 60 °C before further use. The DE-Fc powder within an alumina crucible was placed in a horizontal-wall quartz tube furnace (Lenton). Before starting, the furnace was sealed and fluxed with argon gas (50 sccm) to remove oxygen, and the temperature was raised at a constant rate of 4 °C/min up to the growth temperature of 750 °C. During the synthesis, acetylene (30 sccm), hydrogen (15 sccm), and argon (15 sccm) were fluxed into the furnace for 30 min at standard ambient pressure (760 Torr). At the end of the process, acetylene and hydrogen were stopped, while argon was fluxed for another 15 min to allow it to cool down. The final product (CNT/DE) was then collected from the alumina crucible. 

### 2.3. Preparation of CNS Host by CVD

The synthesis of CNSs was performed following a CVD process carried out in a horizontal hot-wall quartz tube furnace (Lenton). Before starting, the quartz tube was pumped out to remove air (10^−2^ Torr), and the temperature was then raised at constant rate 4 °C/min up to 750 °C. When the system reached this temperature, the pumping was stopped, and argon gas was fluxed until the standard ambient pressure (760 Torr) was restored. Fc catalyst (280 mg) was dissolved in 12 mL dioxane and stirred for 10 min. The solution was then loaded in a syringe and injected into the hot area at a constant rate of 5.6 mL/h under a flux of argon (200 sccm), acetylene (40 sccm) and hydrogen (100 sccm). The synthesis was carried out for two hours. At the end of the process, argon was fluxed (30 sccm) for 15 min to induce cooling down. The final product was then collected from the quartz tube.

### 2.4. Form-Stable PCMs by Impregnation

**Figure 1:**
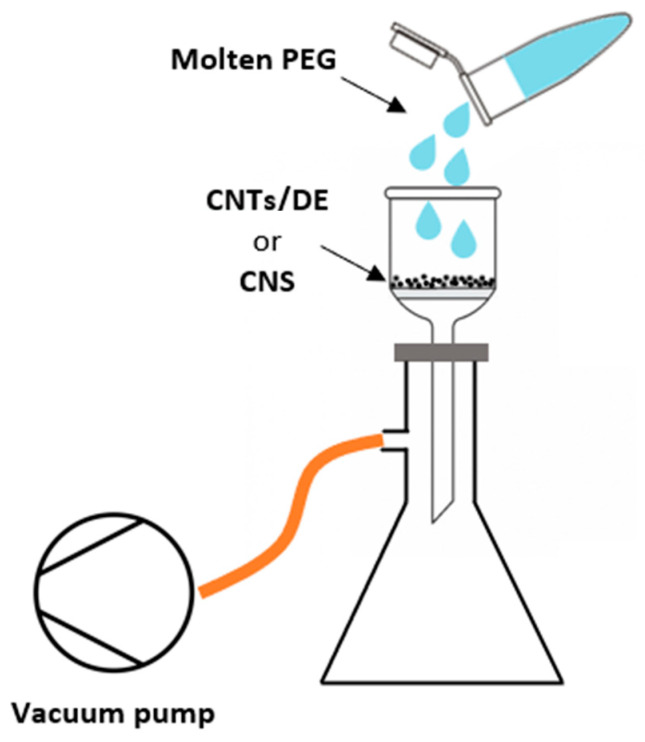
PEG/CNT/DE and PEG/CNS impregnation system.

PEG/CNT/DE and PEG/CNS form-stable PCMs were obtained by impregnation as follows. PEG (2 g) inside a Falcon conical tube was placed in a water bath kept at 40–50 °C until the polymer completely melted. Then, the liquid PEG was quickly poured onto the CNT/DE powder (200 mg) and CNS (100 mg) spread on filter paper placed on top of a Buchner funnel inserted in a vacuum flask, while a vacuum was applied with a rotary pump, as displayed in Figure 1. 

The impregnated materials that were retained by the filters were transferred to a Petri dish, kept at room temperature until the complete solidification of the PEG. Finally, to remove the PEG not entrapped in the porous hosts, both impregnated materials were put in an oven at 60 °C on a filter paper until melt leakage was observed (Figure S1), and then they were kept at room temperature until the complete solidification of the PEG, before being stored in a fridge at a temperature of 4 °C. 

### 2.5. Characterization of the Porous Supports for PCMs: CNT/DE and CS

The morphology of the host structures was investigated using scanning electron microscopy (SEM) (field emission-SEM, LEO Supra 35, Zeiss, Oberkochen, Germany). For this purpose, CNT/DE and CNS samples were previously gold-coated by sputtering (EMITECH K550X sputter coater, Quorum Technologies Ltd., Laughton, East Sussex, UK). The diameter distribution and average diameter of the CNTs grown on DE and composing the CNS were estimated by randomly selecting about 50 CNTs from the acquired SEM micrographs (ImageJ, NIH, Bethesda, MD, USA). The obtained diameter distribution was fitted with two Gaussian functions.

X-ray photoelectron spectroscopy (XPS) spectra were collected on a CNT powder obtained using the same experimental conditions adopted for the CVD growth of CNTs on DE and on a small piece of bare CNS in order to study their surface chemistry, structure, and purity. Both samples were fixed on a Molybdenum sample holder with silver paint and introduced into a chamber operating under ultra-high vacuum conditions (base pressure below 10^−10^ bar). The X-ray data were collected with a semi-imaging analyser MAC 2 (Riber Instruments) operating in the constant pass energy mode (with a total energy resolution of 1.1 eV), using non-monochromatized Al Kα (1486.6 eV) radiation source (10 kV, 10 mA). The distance between the sample and the anode was about 40 mm, the illumination area was about 5 mm ×·5 mm, and the take-off angle between the sample surface and the photoelectron energy analyser was kept fixed at 45°.

Survey (full-range) and high-resolution spectra for C were acquired. Typically, 10 scans were accumulated for each spectrum acquired on three different regions of the two samples. Fitting and deconvolution of the spectra were performed with the help of software developed by MS. The spectra were analysed using a standard Gaussian curve fit routine with a Shirley background subtraction, and the quality of the fit was evaluated using an χ^2^ minimization test. All binding energies were referenced to C(1s) at around 284.6 eV.

An X-ray diffraction (XRD) test was conducted on the CNT/DE powder to analyse the crystalline phase of the sample. The XRD pattern was recorded in the 2θ range 10–60°, adopting a step size of 0.020°, 2 s time per step and scan speed of 0.010°/s, using a Philips X’Pert Pro (Philips N.V, Amsterdam, The Netherlands) diffractometer with Cu Ka radiation (λ = 0.154056 nm). 

The average diameter of CNTs (*τ*) was evaluated from the full width at half-maximum (FWHM) of the diffraction peak at 2θ 26.09°, corresponding to the {002} family of lattice planes of CNTs, employing Scherrer’s Equation:

(1) $$\tau =\frac{K\lambda }{\beta \;cos\theta }$$

where *K* is the shape factor equal to 0.9, *λ* is the wavelength of the Cu Ka radiation, *β* is FWHM (in radians), which is corrected for the instrumental broadening, and *θ* is the Bragg angle.

The thermal behaviour and CNTs weight percentage of the hybrid CNT/DE powder were evaluated by S Measurements were performed under the following conditions: sample weight ~10 mg, air atmosphere, heating rate 10 °C/min up to the peak temperature 900 °C, and dwell time at 900 °C for 10 min. 

The electric measurements were carried out both on the spare CNT/DE powder deposited in a container equipped with four electrical contacts (see Figure S2 in Supplementary Materials) and on a cylindrical pellet (radius 5 ± 1 mm and height 300 ± 50 mm) obtained by pressing the same powder at 5 T for 20 s (see Figure S2). In order to evaluate the specific resistivity of the sample, I–V curves were acquired with a four-probe method with a multimeter (Keithley 2602, Solon, OH, USA). Details of the measurements are reported in the Supplementary Materials.

### 2.6. Characterization of Form-Stable PCMs: PEG/CNT/DE and PEG/CNS

TGA, to evaluate the thermal stability, was performed on the form-stable PCMs (~2 mg) from 25 to 700 °C at 10 °C/min, in air flow (40 mL/min) (Perkin Elmer Pyris 1 TGA) to assess the percentage of the impregnated PEG. Differential scanning calorimetry (DSC) (Netzsch DSC 214 Polyma, Selb, Germany) was performed on 5 mg samples under a nitrogen atmosphere (40 mL/min) using a heating and cooling rate of 10 °C/min in the thermal cycle: heating from −40 °C to 70 °C, cooling from 70 °C to −40 °C. One hundred consecutive heating–cooling cycles were carried out to determine thermal reliability of the developed composites (i.e., PEG/CNT/DE and PEG/CNS) in terms of melting temperatures and latent heats. 

Form-stable PCMs were tested with Fourier-transform infrared spectroscopy (FT-IR) (Cary 630 FTIR, Agilent, Santa Clara, CA, USA) to investigate the chemical interaction between the PCM and the hosts. The analyses were performed in the wavenumber range 4000–600 cm^−1^, with 4 cm^−1^ resolution, and each spectrum was averaged over 32 scans.

The shape-stabilized PCM morphology and the effectiveness of the impregnation with PEG was assessed by SEM analysis.

### 2.7. Analytical Model for Passive Cooling Application in Buildings

**Figure 2:**
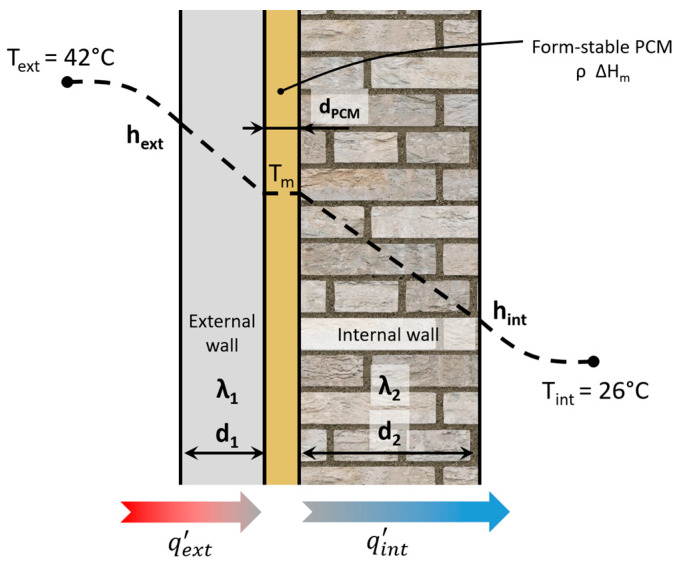
Schematics of the simplified analytical model of a building wall with PCM passive cooling.

The potential performance of the form-stable PCMs in a building’s passive cooling application was evaluated via an analytical heat transfer model. In particular, a building’s exterior wall was modelled as a one-dimensional series of the following layers (with unit area of 1 m^2^): (i) exterior ambient air with a temperature $T_{ext}$ at infinity and convection coefficient $h_{ext}$; (ii) an exterior plaster wall with thickness $d_{1}$ and thermal conductivity $k_{1}$; (iii) a form-stable PCM layer with thickness $d_{PCM}$, melting temperature $T_{m}$, density ρ, and latent heat of fusion $\Delta H_{m}$; (iv) an internal brick wall with thickness $d_{2}$ and thermal conductivity $k_{2}$; and (v) interior ambient air with temperature $T_{int}$ at infinity and convection coefficient $h_{int}$. The analysed system is represented in the schematic in Figure 2, while all the used parameters and their values assumed as input are presented in Table S1 [43,44].

The heat transfer at steady state during the melting transition of the form-stable PCM was analysed by modelling the PCM layer as a zero-dimensional lumped-parameter system that evolves isothermally at temperature $T_{m}$ by exchanging heat with the adjacent layers. With the external air, internal air and PCM temperatures fixed, the power per unit area $q_{ext}^{\prime }$ transferred from the exterior wall to the PCM layer can be computed as in Equation (2), where $R_{ext}$ is the equivalent thermal resistance of the series external air + exterior wall. Similarly, the power per unit area exchanged with the interior wall $q_{int}^{\prime }$ is computed as in Equation (3).

(2) $$q_{ext}^{\prime }=\frac{\left(T_{ext}-T_{m}\right)}{R_{ext}}=\left(T_{ext}-T_{m}\right){\left(\frac{1}{h_{e}}+\frac{d_{1}}{\lambda_{1}}\right)}^{-1}$$

(3) $$q_{int}^{\prime }=\frac{\left(T_{m}-T_{int}\right)}{R_{int}}=\left(T_{m}-T_{int}\right){\left(\frac{d_{2}}{\lambda_{2}}+\frac{1}{h_{i}}\right)}^{-1}$$

In the considered summer case, the form-stable PCM layer absorbs heat $q_{ext}^{\prime }$ from outside and partially releases $q_{int}^{\prime }$ to the inside; the difference between the two values represents the latent heat needed for the phase transition. The energy balance is presented in Equation (4) (where *A* is the wall area, *M* is the *PCM* total mass, and Δt is the time needed for the complete melting of the layer).

(4) $${q^{\prime }}_{PCM}=\left(q_{ext}^{\prime }-q_{int}^{\prime }\right)=\frac{1}{A}\left(M\frac{\Delta H_{m}}{\Delta t}\right)=\rho d_{PCM}\frac{\Delta H_{m}}{\Delta t}$$

By using the experimental values for the thermal and density properties of the form-stable PCMs and assuming a melting time of 12 h, the model outputs can be calculated, e.g., (i) the PCM layer thickness and (ii) the net power transferred to the inside building, i.e., the cooling power needed to maintain the internal temperature. Comparing the latter value with the heat transfer without the presence of the PCM layer, the energy saved can be evaluated. 

## 3. Results

### 3.1. CNT/DE and CNS Porous Hosts for PCMs: Microstructural, Chemical and Thermal Characterization

**Figure 3:**
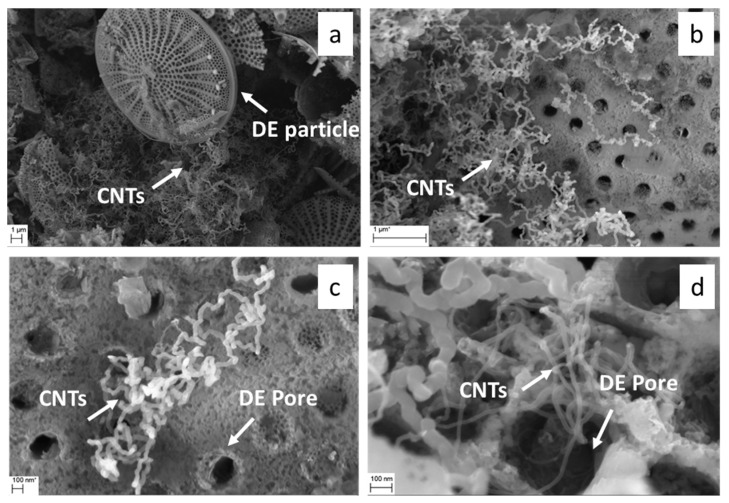
Scanning electron microscopy (SEM) micrographs of CNT/DE sample at low (**a**,**b**) and high magnification (**c**,**d**); scale bars are reported in the images.

The microstructure of the CVD-derived porous hosts was investigated at the microscale by SEM. Representative micrographs of CNT/DE are reported in Figure 3.

**Figure 4:**
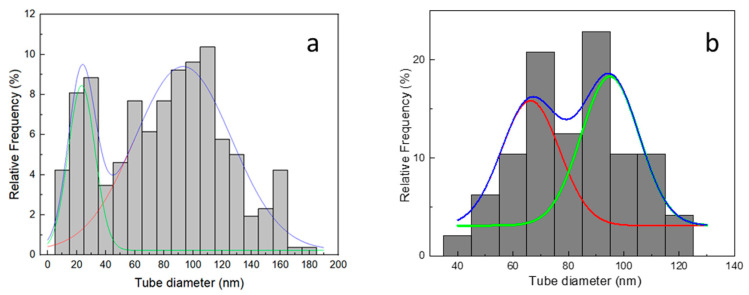
(**a**) CNT diameter distribution of CNT/DE sample from SEM analysis. The blue line is the sum of the two Gaussian curves (red and green lines) and (**b**) CNT diameter distribution of CNS sample from SEM analysis.

The DE host was formed by micrometric fragmented frustules with different sizes and morphologies, belonging to a variety of different species of both centric and pennate diatoms. A wide range of pore diameters spanning from 30 nm to 500 nm were observed on the frustule fragments (Figure 3). The performed CVD process resulted in CNT growth on both the outer surface and inside the porous structure of the frustules. Some zones of the CNT/DE sample show a high quantity of entangled CNTs (Figure 3b), and often, the CNTs originate from inside the DE pores (Figure 3c,d). Presumably, the Fc catalyst was more effectively added in these parts of the sample during the wet method (see Section 2.2). A more uniform CNT coverage is desired to enhance the thermal properties and the specific surface area of the host material. This issue could be addressed by performing surface functionalization of the DE powder and by modifying the process parameters (i.e., time and temperature). A bimodal CNT diameter distribution obtained from a Gaussian fit, centred at 23 ± 9 nm and 93 ± 33 nm, was estimated from the statistical analysis of several SEM images with the same magnification (Figure 4a). 

**Figure 5:**
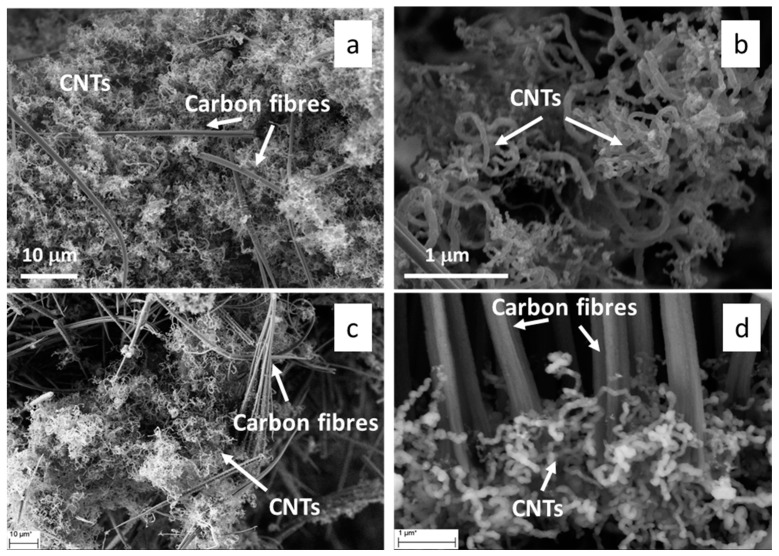
Scanning electron microscopy (SEM) micrographs of a CNS sample obtained a low (**a**,**c**) and high magnification (**b**,**d**); scale bars are reported in the images.

SEM images of the CNS sample are reported in Figure 5. In particular, Figure 5a,c show the presence of CNTs and a lesser extent of carbon fibres. Most of the tubes are not straight but bent and interconnected, as better evidenced in Figure 5b,d. The tube diameter is variable, and a statistical analysis performed from several SEM images at the magnification of Figure 5b points to a double diameter size distribution centred around 66 ± 12 nm and 95 ± 10 nm (Figure 4b).

It is important to highlight that the networks of entangled carbon nanotubes result in a stable self-sustained three-dimensional structure that is highly porous and conductive. The bulk density of the CNS ranges between 10 and 20 mg/cm^3^, while the porosity is estimated to be >96% based on calculations using a density of 2.1 g/cm^3^ for MWCNTs [45]. All these properties can be exploited to directly use the material as a supporting matrix for the confinement of PCMs.

**Figure 6:**
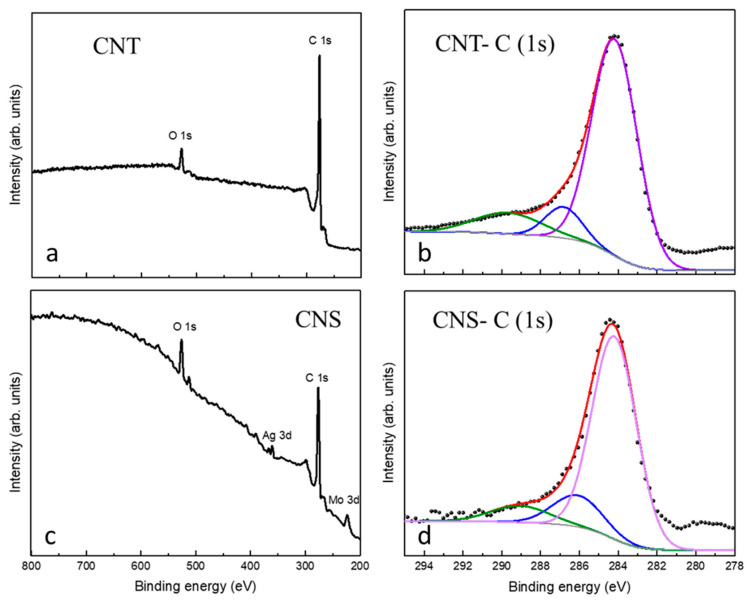
XPS spectra collected on the CNTs and CNS samples. Survey scan for the CNTs in (**a**) and for the CNS sample in (**c**) show the C 1s and O 1s peaks. XPS C1s peak deconvolution for CNT (**b**) for CNS (**d**) samples.

Figure 6 reports the XPS results obtained for the as-grown samples (kept in air) of CNTs (a,b) and of CNS (c,d). The full-range spectra for the CNTs (Figure 6a) and for the CNS sample (Figure 6c) show the C 1s and O 1s peaks. In the CNT spectrum, the Mo 3p and the Ag 3d peaks originate from the sample-holder and silver paint used to fix the sample.

The experimental C1s XPS spectrum reported in Figure 6b was analysed to investigate the presence of surface functional groups on the CNTs. The C1s peak can be deconvoluted into three main components: 284.6 eV sp^2^ carbon phase C-C (79.6%), C-O and C=O bonds (hydroxyls and carbonyls) at 286.8 eV (9.2%), and -COOH bond (carboxyles) at 289.8 eV (11.3%) [46] Also, the XPS analysis on the CNS sample gave similar results, pointing to a partial oxidation of the carbon nanomaterial. In particular, the C 1s spectrum fit analysis (Figure 6d) gave three main contributions: 284.2 eV sp^2^ carbon phase C-C (78%), C-O and C=O bonds at 286.0 eV (13.7%), and -COOH bond at 289.0 eV (8.3%) [46].

**Figure 7:**
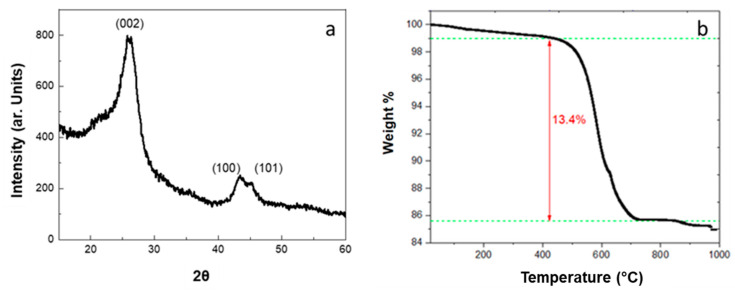
X-ray diffraction pattern (**a**) and TGA curve (**b**) of CNT/DE hybrid powder.

The structural characterization of CNTs grown on diatomite was performed by XRD. The XRD pattern of the hybrid powder shows the characteristic peaks of multi-walled carbon nanotubes (MWCNTs) at 2θ = 26.09°, 43.58° and 45.11° due to (002), (100) and (101) reflections, respectively (Figure 7a) [47]. From XRD data, an average diameter of MWCNTs of 2.3 nm was evaluated, consisting of six to seven concentric graphene layers spaced 0.3413 nm apart. In the XRD pattern, there is no contribution that can be ascribed to silica crystalline phases of diatomite. 

The characterization of CNT/DE powder was further pursued using TGA (Figure 7b). The thermogram shows two weight-loss steps: (i) RT–430 °C (−1 wt.%) due to the removal of adsorbed water and the decomposition of amorphous carbon and (ii) 430–730 °C (−13.4 wt.%), associated with the decomposition of the nanotubes, in accordance with the literature [48]. Since DE is thermally stable in the temperature range of the measurement, and considering the first weight loss (−1 wt.%), the weight ratio CNT/DE is 0.16. 

**Figure 8:**
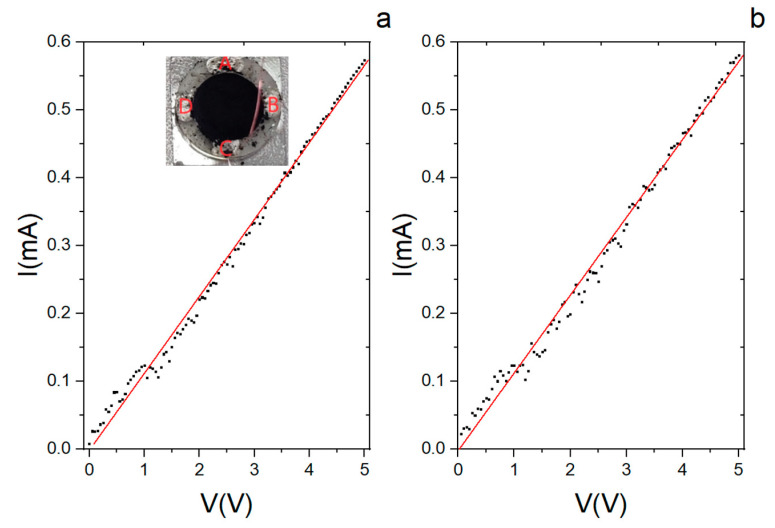
Current–voltage (I–V) curves from the CNT/DE powder. (**a**) The voltage between contacts A and B is first varied, and the current between contacts C and D is measured. (**b**) The voltage between B and C is varied, measuring the current between D and A. Black dots are the experimental data, and red lines are the curve fits. Inset: set-up for the four-contact measurement.

The decoration of diatomite with CNTs also gave an important contribution to the electric behaviour of the hybrid system. An electrical characterization of the system was performed by acquiring the current–voltage response (I–V curve) following the four-probe method of van der Pauw (see Supplementary Materials for details Figure S3, as well as Figure 8) [49]. The measurements were at first carried out directly on the powder put in a special sample holder equipped with four electrical contacts, as shown in the inset of Figure 8a. During the test, the voltage was first applied between the A and B contacts while acquiring the current signal between the contacts C and D (Figure 8a); then, the voltage was applied between B and C while measuring the current between D and A (Figure 8b). A linear response was observed in both measurements (black dots), and from the analysis (red lines), resistance values equal to 8.81 ± 0.07 kΩ and 8.71 ± 0.08 kΩ were obtained for the data of Figure 8a and 8b, respectively. It can be noticed that although there are fluctuations in the current signal, the system had, overall, a linear trend. From the SEM analysis (see Figure 2), the CNTs are not always in close contact with one another, but their distribution in the matrix is sufficient to give an electrical response, as confirmed by the similarity of the two obtained curves. 

**Figure 9:**
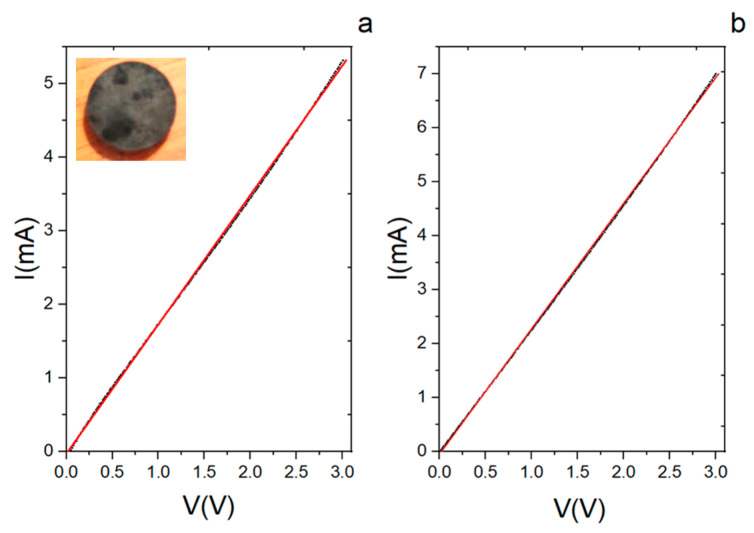
Current–voltage (I–V) curves from the tablet made from the CNT/DE powder after being pressed at 5 T for 20 s. (**a**,**b**) were obtained by applying the voltage and measuring the current, as in the case of the powder. Black dots are the experimental data and red lines are the curve fits. The tablet used for the van der Paw measurement is reported in the side picture.

The same electrical measurement following the four-probe method of van der Pauw was applied on a tablet obtained from the hybrid material after being pressed at 5 T for 20 s. The I–V curves are reported in Figure 9 (the black dots are experimental data, and the red line is the curve fit) together with a picture of the pellet. 

Both curves have the same linear trend, and from the analysis, the calculated resistance is 568 ± 1 Ω and 430 ± 1 Ω from the curves in Figure 8a and Figure 7b, respectively. The linear response is also confirmed in this case. The conductive response has to be assigned to the presence of the CNTs in the otherwise insulating diatomite. The linear response for the powder or the pellet points to an Ohmic behaviour with a difference in the obtained resistance value of one order of magnitude between the two samples. This result was somehow expected since, in the pellet, the number of contacts between the tubes in the mesh was remarkably higher due to the compact structure obtained after the compression. The relationship between the variation in the conductance and the number of contacts as a function of the applied pressure has been described theoretically [50] and verified experimentally for assemblies of CNTs [51].

### 3.2. PEG/CNT/DE and PEG/CNS Form-Stable PCMs: Thermal Properties and Microstructure

**Figure 10:**
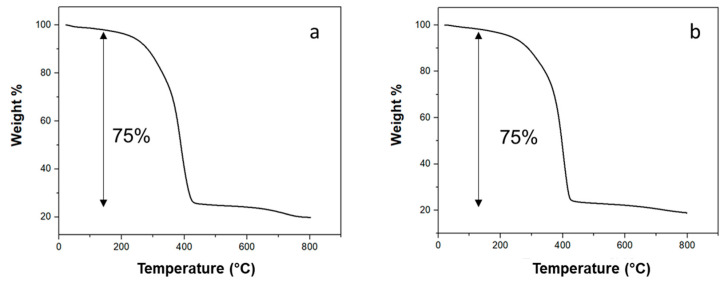
TGA curve of PEG/CNT/DE (**a**); TGA curve of PEG/CNS (**b**).

TGA curves of PEG/CNT/DE and PEG/CNS samples are presented in Figure 10a and 10b, respectively. Weight loss at temperatures of up to 430 °C for both form-stable PCMs (Figure 10a,b) can be associated with PEG decomposition [52], while the weight loss observed for PEG/CNT/DE and PEG/CNS samples at T > 500 °C has to be attributed to the decomposition of the CNTs decorating DE and to the carbon-based materials (CNTs and carbon fibres) constituting the sponges, respectively. Therefore, the amount of impregnated PEG is 75 wt% for both form-stable PCMs. Regarding the PEG/CNT/DE sample, the value is slightly higher with respect to that reported in the literature for neat diatomite employed as a host for PEG, ranging between 41% and 70% [37,53], presumably due to the presence of the CNTs increasing the specific surface area of the DE-based host.

**Figure 11:**
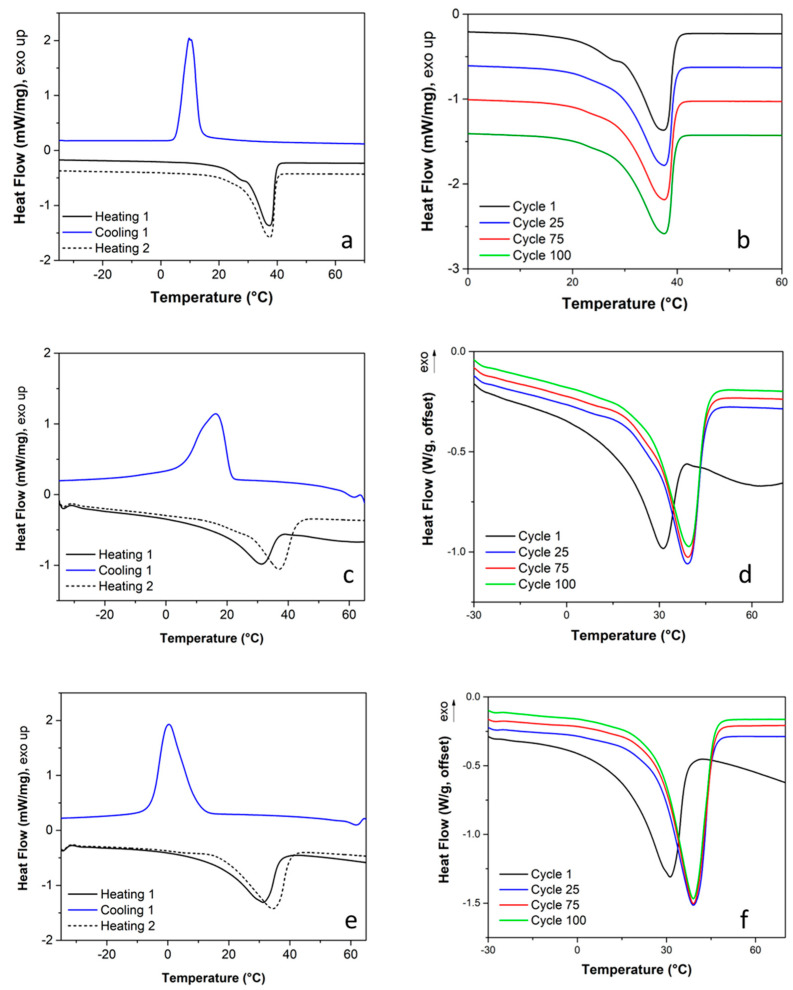
DSC heating and cooling of PEG (**a**), PEG/CNT/DE (**c**) and PEG/CNS (**e**). Melting region of cyclic DSC of PEG (**b**), PEG/CNT/DE (**d**) and PEG/CNS (**f**).

The thermal energy storage properties, such as melting temperature and latent heat value, of pure PEG, PEG/CNT/DE and PEG/CS samples were determined by DSC (Figure 11a–e and Table 1).

As seen in the DSC heating curves in Figure 11a,b, neat PEG crystalline regions melt in the temperature range between 15 °C and 45 °C, as indicated by the presence of endothermic peaks. In the first heating, two endothermic peaks are clearly visible at 29.5 °C and 37.6 °C. 

The presence of multiple endothermic peaks is associated with a non-uniform size distribution of crystallites. In the subsequent heating curves, only one endothermic peak located at 37.3 °C, preceded by a low-intensity shoulder, is observed. These results suggest an improvement in the size homogeneity of crystalline regions induced by the first thermal cycle. The cooling curve shows an exothermic peak associated with crystallization (Figure 11a). The latent heat that PEG can store during consecutive solid–liquid phase transitions is high and almost constant (122 ± 1; see Table 1), making it a high-yield phase-change material.

The endothermic signals of the DSC heating curves for PEG/CNT/DE and PEG/CNS composites (Figure 11c,d and Figure 10e,f, respectively) associated with the melting of PEG fall in the temperature range between 15 °C and 43 °C. For both form-stable PCMs, a first-melting endothermic peak at 31.2 °C is present. First-heating melting temperatures for PEG/CNT/DE and PEG/CNS (31.2 °C) composites are very close to the first endothermic peak of neat PEG (29.5 °C) and are a direct consequence of rapid cooling during the impregnation process. Indeed, carbon nanostructures can behave as nucleating agents, allowing for the formation of low-melting thinner lamellae [54]. Moreover, their presence can modify the thermal conductivity, leading to different crystallization kinetics [55]. Such results suggest that after manufacturing, the crystallite sizes of composite PCMs are comparable to those of neat PEG. After the first five cycles, both form-stable PCMs display a peak of the melting region at around 39 °C. The composite systems keep the latent heat accumulation capacity upon 100 melting–solidification cycles (81.5 ± 5.3 J/g and 108.2 ± 4.6 J/g, for PEG/CNT/DE and PEG/CNS, respectively) (see Table 1 and Figure S4). As expected, the melting enthalpy values were lower compared to those of neat PEG due to the presence of the host materials (CNT/DE and CNS). Moreover, DSC results show that both host materials do not significantly influence the melting temperature range of PEG and keep the reproducibility of PEG melting over the thermal cycles, as shown in Figure 11d,f. 

**Figure 12:**
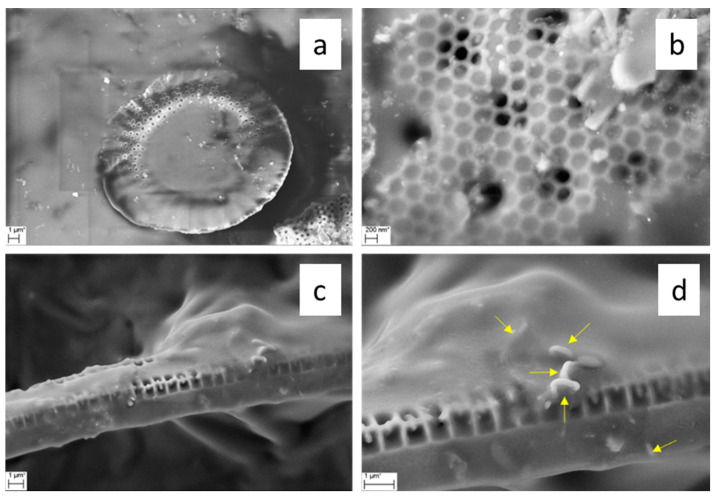
SEM micrographs of form-stable PEG/CNT/DE at (**a**) low magnification (**b**) high magnification. Presence of CNTs is visible in (**c**) and highlighted in (**d**) where arrows indicate the CNTs.

**Figure 13:**
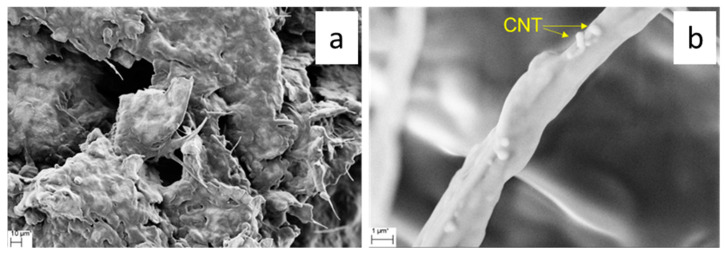
SEM micrographs of form-stable PEG/CNS PCM at low magnification (**a**) and high magnification (**b**) where arrows indicate the CNTs.

SEM images of the form-stable PCMs are presented in Figure 12 and Figure 13.

SEM analysis showed a good adhesion between PEG and the CNTs–decorated frustules, which were completely coated by the polymer (Figure 12a–d). Moreover, PEG penetrated inside the hierarchal pore structure, also filling the nanometric holes of frustules, as shown in Figure 12b. 

Also, for the PEG/CNS sample, the PCM well coated the porous support (Figure 13a,b). PEG adhered well to the interconnected structure of the sponge composed of CNTs and carbon fibres. These results indicate a good compatibility of the PCM with both the support matrices, and that the impregnation process carried out was efficient. 

**Figure 14:**
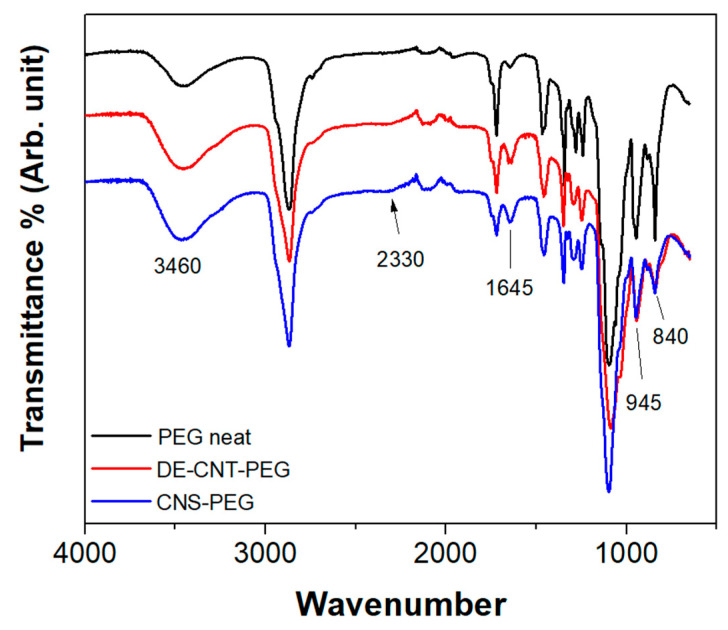
FT-IR of neat PEG and shape-stabilized PEG–host samples.

The interaction between PCMs and the hosts was investigated via FT-IR analysis, the results of which are reported in Figure 14.

For the three samples, the characteristic PEG absorption bands were identified as follows: C-H stretching at 2875 cm^−1^; CH_2_ scissoring at 1467 cm^−1^; CH_2_ wagging at 1342 cm^−1^; CH_2_ twisting at 1242 and 1280 cm^−1^; C-O and C-C stretching at 1095 cm^−1^; C-O and C-C stretching and CH_2_ rocking at 840 cm^−1^; and CH_2_ rocking and CH_2_ twisting at 945 cm^−1^ [56]. The increase in the intensity of the bands at 3460 cm^−1^ observed for the composite PCMs with respect to the neat PEG, corresponding to O-H stretching (hydroxyls, moisture), can be ascribed to the presence of partially oxidated CNTs, in line with the findings of the XPS measurements. Moreover, the presence of a sharp peak at 1720 cm^−1^ (C=O stretching) for all the three investigated samples further confirms a partial oxidation, while the increase in intensity of the band at 1645 cm^−1^ ascribed to both the O-H bending and C=C stretching mode of the aromatic ring is due to CNT presence. 

It is worth highlighting that the presence of a broad peak at 2330 cm^−1^ related to the O-H stretching mode from hydrogen-bonded carboxyl groups can be associated with the bonding occurring between carboxyles of CNTs and the ether oxygen (–O–) of PEG, which contributes to the shape stabilization mechanisms of PCM in the hosts [57]. 

### 3.3. Performances of PCM in Building Passive Cooling Application

The proposed analytical model allowed for a simplified estimation of the performances of the form-stabilized PCM materials as a passive cooling layer in buildings in extreme summer temperatures. Considering an outside temperature of 42 °C, the calculated power per unit area transferred through the wall without PCM to maintain an inside temperature of 26 °C was 10.06 W/m^2^, which corresponds to the cooling power needed to maintain the inside temperature. By applying a layer of form-stable PCM, part of the transmitted energy was absorbed by the material during its melting process, which involved an average melting enthalpy of 82.6 kJ/kg and 109.2 kJ/kg for the PEG/CNT/DE and PEG/CNS, respectively, as calculated from the DSC curves. With this layer, the transmitted power by unit area decreased to 9.35 and to 9.07 W/m^2^ for the PEG/CNT/DE and PEG/CNS, respectively, representing a reduction in required cooling power of about 10%. Moreover, it was calculated that the layer thickness needed to obtain a time for complete melting of 12 h was ~2.5 mm for both materials. 

It must be noted that the employed model represents a worst-case scenario, both regarding the environmental boundary conditions, which are set to a fixed 42 °C of outside temperature, and regarding the simplified thermal model. Indeed, the model assumes that the PCM phase change happens isothermally at the measured DSC melting peak temperature, while in reality, it takes place in the range 17–48 °C. Thus, the PCM layer would start to melt at lower temperatures than the T_m_ assumed by the simplified model, and therefore, it would isolate the inside environment more efficiently, reducing the required cooling power by more than the computed 10%. The form-stable PCMs, therefore, could represent an effective sustainable solution to reduce the power needed for the thermal management of buildings, aiding in the decrease in CO_2_ emissions, which is paramount to limit global warming.

## 4. Conclusions

Incorporating form-stabilized PCMs into building envelopes is a booming technology to improve indoor thermal comfort and, consequently, reduce energy consumption in buildings. In this study, two types of porous supporting materials consisting of CNTs–decorated diatomite (CNT/DE) and CNT sponges (CNS) were developed, following a CVD simple route, to prepare novel form-stable PCM composites by impregnation, using PEG as the PCM. SEM analysis of the CNT/DE support matrix showed highly entangled nanotubes over the outer surface and inside the porous structure of diatomite, with a bimodal diameter distribution centred at 23 nm and 93 nm. The weight ratio CNT/DE evaluated by TGA is 0.16, which is enough to give the hybrid matrix an electrical response, as evidenced by the measurements performed following the van der Pauw method.

The resulting CNS was mainly composed of bent and interconnected CNTs, with a double diameter distribution centred around 66.3 nm and 95 nm, forming a three-dimensional, highly porous structure. The presence of oxygen-containing functional groups was detected for both developed host materials. 

Due to their high porosity, both hosts were guaranteed to have high loads of PEG, about 75 wt% as evaluated by TGA, without PCM leakage during melting, forming stable PCM composites with homogeneous morphology as assessed by SEM analysis.

DSC results for both form-stable PCM composites revealed a high thermal reliability upon several melting–solidification cycles in terms of the latent heat (86 ± 4 J/g and 100 ± 2 J/g for PEG/CNT/DE and PEG/CNS samples, respectively) and melting temperature range of PEG (between 15° and 45°). An analytical model was used to simulate the thermal behaviour of a building wall containing the PCM confined in the developed hosts. A reduction of 10% in the transmitted thermal power was obtained with a form-stable PCM layer thickness of 2.5 mm. The overall results suggest that the developed form-stable PCMs composites are promising candidate materials to be employed in thermal energy storage application.

## Figures and Tables

**Table 1:** Melting enthalpies calculated by the areas subtended to the endothermic peaks between 15 °C and 45 °C of the DSC heating curves of PEG, PEG/CNT/DE, and PEG/CNS.

	PEG	PEG/CNT/DE	PEG/CNS
**Cycle 1**	122.31 J/g	74.09 J/g	78.88 J/g
**Cycle 25**	122.11 J/g	82.71 J/g	108.60 J/g
**Cycle 75**	123.09 J/g	82.73 J/g	109.81 J/g
**Cycle 100**	122.94 J/g	82.82 J/g	110.43 J/g

## Data Availability

Data will be available on request.

## Supplementary Materials

The following supporting information can be downloaded at: https://www.mdpi.com/article/10.3390/ma17235721/s1, Figure S1: Leakage test at 60 °C (a) for PEG/CNTs/DE (b) and PEG/CNS (c); Table S1: Parameters used in the analytical model and their assumed values; Figure S2: Pictures of the two samples used for the electrical measurements on the diatomite-CNT powder and a schematic of the four-probe method. Figure S3: Van der Pauw function for the diatomite-CNT powder (a) and for the tablet (b). Figure S4: DSC Thermal cycles for (a) PEG/CNTs/DE and for PEG/CNS (b) samples.
