# Supplementary material for: Form-Stable Phase-Change Materials Using Chemical Vapor Deposition-Derived Porous Supports: Carbon Nanotube/Diatomite Hybrid Powder and Carbon Nanotube Sponges
Source: Materials (Basel). 2024 Nov 22;17(23):5721. doi: 10.3390/ma17235721 (PMC11642659; doi:10.3390/ma17235721)
Supplement: Supplementary file 1 [file materials-17-05721-s001.zip › materials-3310216-supplementary.pdf]

Supplementary Materials

Form-Stable Phase-Change Materials Using Chemical Vapor Deposition-Derived Porous Supports: Carbon Nanotube/Diatomite Hybrid Powder and Carbon Nanotube Sponges

Francesca Romana Lamastra[1], Mario Bragaglia [1,*], Lorenzo Paleari[1], Francesca Nanni[1], Francesco Fabborcino[2], Manuela Scarselli[3]

[1] Università degli Studi di Roma "Tor Vergata", Dipartimento di Ingegneria dell'Impresa "Mario Lucertini" and Consorzio INSTM Unità di Ricerca "Roma Tor Vergata", Via del Politecnico, 00133, Roma, Italy;
lamastra@scienze.uniroma2.it (F.R.L.); lorenzo.paleari@uniroma2.it (L.P.); fnanni@ing.uniroma2.it (F.N.)

[2] Pegaso Telematic University, Department of Engineering, 80143 Naples, Italy; francesco.fabbrocino@unipegaso.it

[3] Università degli Studi di Roma "Tor Vergata", Dipartimento di Fisica, Via della Ricerca Scientifica,
00133 Roma, Italy; manuela.scarselli@roma2.infn.it

* Correspondence: bragaglia@ing.uniroma2.it

Table S1. Parameters used in the analytical model and their assumed values

Domain	Parameter	Symbol	Value
Exterior ambient air	Temperature at infinity	T_{ext}	42 °C
	Convection coefficient	h_{ext}	20 W/m²K
External plaster wall	Wall thickness	d_1	2 cm
	Thermal conductivity	λ_1	0.22 W/mK
Form-stable PCM layer	Layer thickness	d_{PCM}	output
	Melting temperature	T_m	experimental value
	Latent heat of fusion	ΔH_m	experimental value
	Density	ρ	experimental value
	Time for complete melt	Δt	12 h
Internal brick wall	Wall thickness	d_2	20 cm
	Thermal conductivity	λ_2	0.3 W/mK
Interior ambient air	Temperature at infinity	T_{int}	26 °C
	Convection coefficient	h_{int}	8.6 W/m²K

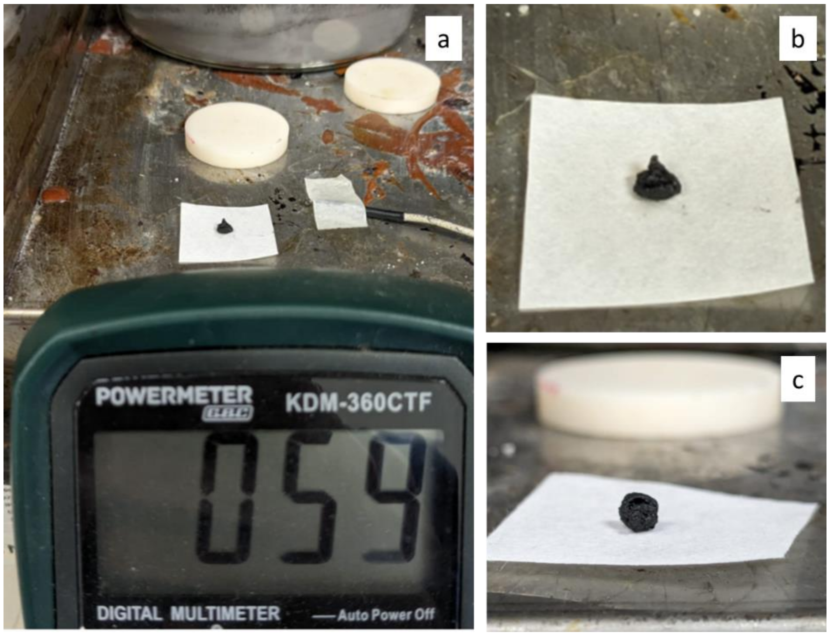

Figure S1. Leakage test at 60 °C (a) for PEG/CNTs/DE (b) and PEG/CNS (c)

Electrical measurements: van der Pauw method

Following Pauw's method, four metal contacts are applied on the sample denoted as A, B, C and D in the Figure S1. The voltage between contacts A and B is first varied and the current between contacts C-D is measured, then the voltage between B-C is varied measuring the current between D-A. From the I-V curves obtained is possible to evaluate the resistance. The same method was also applied to a diatomite-CNT tablet obtained by subjecting the powder to a pressure of 5T for 20 seconds.

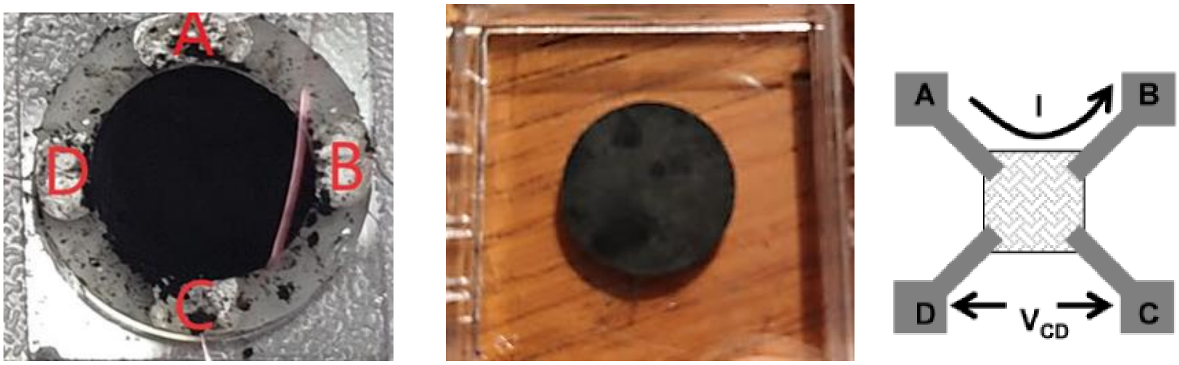

Figure S2. Pictures of the two samples used for the electrical measurements on the diatomite-CNT powder and a schematic of the four-probe method. On the left, the spare diatomite-CNT powder was deposited in a container equipped with four electrical contacts and on the right the powder was pressed at 5 T for 20 s to from a tablet and then measured with the four electrical contacts configuration.

To conclude the electric characterization, we also graphited the van de Pauw function, [1] shown in Figure S3, to evaluate the sheet resistance ρ/d, obtained when the f(ρ/d) = 1 as indicated in the figure. For the powder ρ/d =39.7±0.3 kΩ/sq and for the tablet ρ/d = 2.246±0.005 kΩ/sq. In particular, in the case of the tablet it was possible to measure the thickness (d=0.30±0.02mm) and the resistivity found is ρ= 6.74±1.14 Ωm.

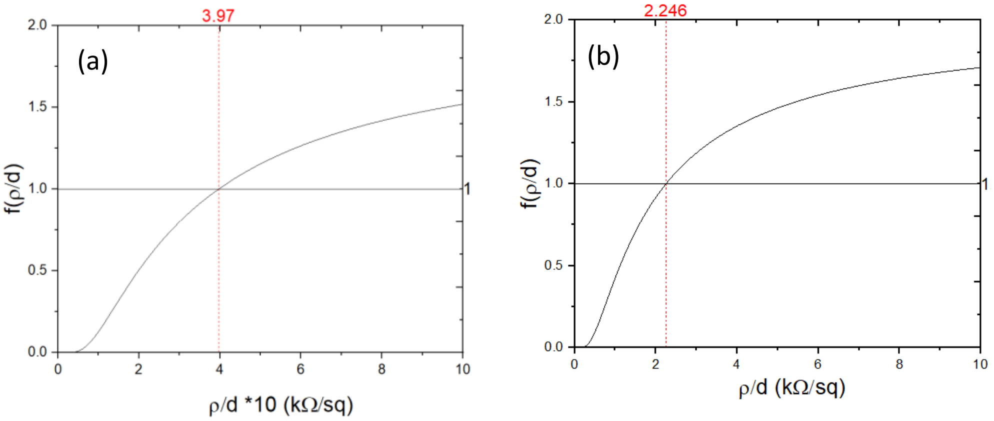

Figure S3. Van der Pauw function for the diatomite-CNT powder (a) and for the tablet (b).

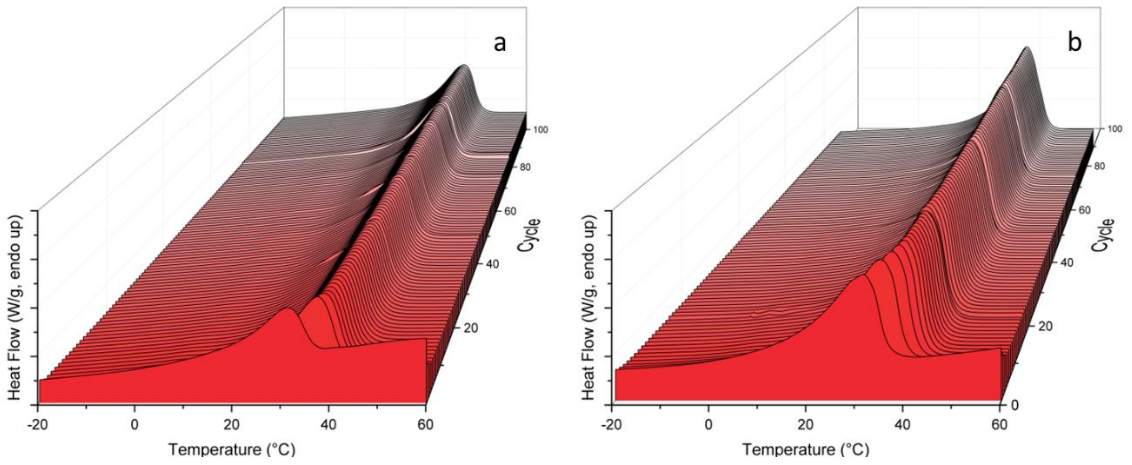

Figure S4. DSC Thermal cycles for (a) PEG/CNTs/DE and for PEG/CNS (b) samples.

REFERENCES

[1] The van der Pauw function can be expressed as $f(\frac{d}{\rho}) = e^{-\left(\pi R_{AB,CD} {}^{d}/_{\rho}\right)} + e^{-\left(\pi R_{BC,DA} {}^{d}/_{\rho}\right)}$, where $R_{AB,CD}$ and $R_{BC,DA}$ are the values of the electrical resistance estimated from the four probe measurement, d is the sample thickness and ρ is the resistivity. From ref. A method of measuring specific resistivity and Hall effect of discs of arbitrary shape, L. J. van der Paw, Philips Res. Reports 13, 1-9, (1958).
